# Correlation of Plasma Membrane Microviscosity and Cell Stiffness Revealed via Fluorescence-Lifetime Imaging and Atomic Force Microscopy

**DOI:** 10.3390/cells12212583

**Published:** 2023-11-06

**Authors:** Yuri M. Efremov, Liubov Shimolina, Alexander Gulin, Nadezhda Ignatova, Margarita Gubina, Marina K. Kuimova, Peter S. Timashev, Marina V. Shirmanova

**Affiliations:** 1 Institute for Regenerative Medicine, Sechenov First Moscow State Medical University (Sechenov University), 119991 Moscow, Russia; timashev_p_s@staff.sechenov.ru; 2 Institute of Experimental Oncology and Biomedical Technologies, Privolzhsky Research Medical University, 603005 Nizhny Novgorod, Russia; shimolina_l@pimunn.net (L.S.); ignatova_n@pimunn.net (N.I.); shirmanova_m@pimunn.net (M.V.S.); 3 N.N. Semenov Federal Research Center for Chemical Physics, Russian Academy of Sciences, 119991 Moscow, Russia; aleksandr.gulin@phystech.edu (A.G.); gubina.mv@phystech.edu (M.G.); 4 Department of Chemistry, Imperial College London, White City Campus, London W12 0BZ, UK; m.kuimova@imperial.ac.uk; 5 World-Class Research Center “Digital Biodesign and Personalized Healthcare”, Sechenov University, 119991 Moscow, Russia

**Keywords:** plasma membrane, actin cytoskeleton, cancer cell, microviscosity, viscoelasticity, molecular rotors, fluorescence-lifetime imaging microscopy (FLIM), time-of-flight secondary ion mass spectrometry (ToF-SIMS), atomic force microscopy (AFM)

## Abstract

The biophysical properties of cells described at the level of whole cells or their membranes have many consequences for their biological behavior. However, our understanding of the relationships between mechanical parameters at the level of cell (stiffness, viscoelasticity) and at the level of the plasma membrane (fluidity) remains quite limited, especially in the context of pathologies, such as cancer. Here, we investigated the correlations between cells’ stiffness and viscoelastic parameters, mainly determined via the actin cortex, and plasma membrane microviscosity, mainly determined via its lipid profile, in cancer cells, as these are the keys to their migratory capacity. The mechanical properties of cells were assessed using atomic force microscopy (AFM). The microviscosity of membranes was visualized using fluorescence-lifetime imaging microscopy (FLIM) with the viscosity-sensitive probe BODIPY 2. Measurements were performed for five human colorectal cancer cell lines that have different migratory activity (HT29, Caco-2, HCT116, SW 837, and SW 480) and their chemoresistant counterparts. The actin cytoskeleton and the membrane lipid composition were also analyzed to verify the results. The cell stiffness (Young’s modulus), measured via AFM, correlated well (Pearson r = 0.93) with membrane microviscosity, measured via FLIM, and both metrics were elevated in more motile cells. The associations between stiffness and microviscosity were preserved upon acquisition of chemoresistance to one of two chemotherapeutic drugs. These data clearly indicate that mechanical parameters, determined by two different cellular structures, are interconnected in cells and play a role in their intrinsic migratory potential.

## 1. Introduction

The biomechanics of cells influences many aspects of cell growth and functioning, including tissue development and homeostasis, interaction with the extracellular matrix, differentiation, migration, division, etc. Mechanical properties change in response to both a cell’s environment and internal transcriptional and translational signals. The main parameters that determine the mechanical properties of eukaryotic cells are the deformability of the cytoskeleton and the fluid-like properties of the plasma membrane [1,2,3].

Methods that deform the cell locally but inflict significant deformations (atomic force microscopy (AFM), cell poking, magnetic twisting cytometry, nanoindentation) usually determine the measured mechanical response to the cytoskeleton, mostly the actomyosin cortex, but ignore the lipid bilayer of the plasma membrane [4,5,6,7]. The lipid membrane is assumed to have low bending rigidity, and the excess membrane area is stored in protrusions and invaginations, such as microvilli [8], that can be pulled out to dilate the membrane area during tension [9]. Tether pulling experiments, in which plasma membrane nanotubes are extracted from the cell using optical tweezers or an AFM cantilever, demonstrated that membrane–cortex interactions account the most for effective membrane tension [10,11,12,13,14]. The cellular cytoskeleton provides a resistive force, which locally opposes the applied tension and prevents the propagation of force through the cell membrane [15,16]. The actomyosin cortex is a thin (100–500 nm) layer of actin, myosin, and actin-binding proteins that underlies the plasma membrane [17] and is linked to the lipid bilayer via specialized proteins, like the proteins of the ezrin, radixin, and moesin (ERM) families [18]. The cortex behaves as a viscoelastic material with pre-tension in it [17,19]. Modifications in the cortex or in the actin cytoskeleton in general (e.g., via specific inhibitors of protein interaction or activities) have a dramatic effect on cell mechanics [20,21,22].

The plasma membrane is a complex of lipids and proteins that ensures the integrity of the cell and controls the interaction between the cell and its environment. From the point of view of modern cell biomechanics, the plasma membrane is a composite material, in which the dynamic relationship between the lipid bilayer and the sub-membrane cytoskeletal cortex determines its mechanical behavior [1]. A fluid state of the membrane plays a critical role in regulation of the membrane’s semipermeability, the arrangement of the membrane proteins, and transmembrane transport. The fluidity of membranes is primarily determined by their qualitative and quantitative lipid composition [23]. Specifically, cholesterol, saturated fatty acids in phospholipids, and sphingomyelin are known to stiffen a membrane [24,25,26]. The sub-membrane cytoskeleton can also affect the viscous properties of the membrane; however, the mechanisms of membrane–cytoskeleton interactions are still poorly understood. It is supposed that these interactions are mediated by the integral membrane proteins that are linked to the cytoskeleton through their intracellular domains and serve as stabilizers of fluid properties [27]. 

The neoplastic transformation of cells unavoidably leads to changes in cellular biomechanics in a way that favors tumor development, invasion, and dissemination in the body. At the cellular level, the majority of cancerous cells are more deformable in comparison to normal cells [28,29]. Cell softening generally agrees with the structural changes in the actin cytoskeleton of cancer cells, which lacks well-developed stress fibers and is more disorganized [20,30]. Several studies have shown that tumor cell membranes have higher fluidity than normal cell membranes [31,32], and their microviscosity depends on the histological origin of the tumor [33,34]. It has been also demonstrated that the accelerated membrane fluidity of cancer cells is closely related to cancer invasiveness and metastases [35,36]. While the role of the cytoskeleton, and especially actin, in relation to the mechanical characteristics of cancer cells has been extensively studied, changes in the viscoelastic properties of the cell membrane have been much less explored.

Although the mechanical properties of both the cortex and membrane are of fundamental importance for the physiological functioning of any living cells, the relationships between cell stiffness and membrane fluidity remain poorly understood, especially in the context of cancer. In the present study, we aimed to investigate the connection between membrane microviscosity and cortex mechanics. We performed complementary measurements on colorectal cancer cell lines with different migratory activity using fluorescence-lifetime imaging microscopy—FLIM (membrane viscosity) and atomic force microscopy—AFM (Young’s modulus, cell-level cortex viscoelasticity). To support the results of AFM and FLIM, we also analyzed the actin cytoskeleton via confocal microscopy and the lipid composition of the plasma membrane via time-of-flight secondary ion mass spectrometry (ToF-SIMS).

## 2. Materials and Methods

### 2.1. Cell Cultures 

The HCT116, HT29, Caco-2, SW 837, and SW 480 (human colorectal adenocarcinoma) cell lines were used in the study. The cells were cultured in DMEM containing 100 μg/mL penicillin, 100 μg/mL streptomycin sulfate, and 10% fetal bovine serum in an incubator at 37 °C in a humidified atmosphere with 5% CO_2_.

Sublines of HCT116 and HT29 cells resistant to the chemotherapeutic drugs oxaliplatin (HCT116-OXAR, HT29-OXAR) and 5-fluorouracil (HCT116-5FUR, HT29-5FUR) were also used.

The resistant cells were obtained previously via continuous exposure to gradually increasing drug concentrations [37]. By ~20 weeks after the first drug exposure, the cells were considered resistant. The resistant cells were cultured in the presence of corresponding drugs, oxaliplatin (5 mg/mL, Teva, Kefar Sava, Israel) or 5-fluorouracil (50 mg/mL, Ebewe Pharma, Unterach am Attersee, Austria). The chemotherapeutic agents were removed from the culture medium 48 h before the experiments in order to avoid possible effects of direct interaction of the drug with the cell membrane [38,39].

### 2.2. Measuring Membrane Microviscosity via FLIM Using a Molecular Rotor

The molecular rotor BODIPY 2 (4,4-difluoro-4-bora-3a,4a-diaza-s-indacene) was used for microviscosity imaging [40]. BODIPY 2 demonstrates the sensitivity of its fluorescence emission to the viscosity of the immediate environment with a good dynamic range of fluorescence lifetimes and applicability in biological samples [41].

Cancer cells were seeded on glass-bottomed FluoroDishes in complete DMEM media without phenol red (Life Technologies, Carlsbad, CA, USA) and incubated for 24 h. Then, staining of the cells with BODIPY 2 was performed according to the protocol described in [42]. The culture medium was replaced with ice-cold Hank’s solution without Ca^2+^/Mg^2+^ ions, and the cells were incubated at +4 °C for 3 min. Then, Hank’s solution was replaced with ice-cold BODIPY 2 solution in phosphate-buffered saline (PBS) (4.5 μM, 0.1% DMSO). FLIM images of the cells were acquired within ~20 min after staining with BODIPY2. At these concentrations, we previously demonstrated that the insertion of the dye has no effect on the properties of the membranes [43].

An LSM 880 (Carl Zeiss, Jena, Germany) laser scanning microscope equipped with an FLIM module, an SPC 150 TCSPC (Becker & Hickl GmbH, Berlin, Germany), and a Mai Tai HP femtosecond laser, 80 MHz, 140 fs (Spectra Physics, Milpitas, CA, USA) were used for FLIM. The two-photon fluorescence of BODIPY 2 was excited at a wavelength of 850 nm and detected in the range 500–550 nm. A C-Plan-Apochromat 40×/1.2 NA objective lens was used for image acquisition. The FLIM images were acquired at a laser power of 1–2%, with a photon collection time of 60 s, to provide ≥5000 photons per decay curve. Examples of the decay curves are presented in Figure S1. Images were obtained from 10 randomly selected fields of view in each dish. 

Fluorescence-lifetime analysis was performed using SPCImage software 8.3 (Becker & Hickl GmbH, Berlin, Germany). The fluorescence lifetimes were analyzed in the plasma membranes of the cells through the manual selection of zones with a reasonable fit as regions of interest. The fluorescence decay curves of BODIPY2 were fitted to a monoexponential decay model. The goodness of the fit, the χ^2^ value, was in the range of 0.8 to 1.2. The experimentally measured lifetimes of BODIPY2 (in ns) were converted to viscosity values (in cP) using a calibration curve obtained previously [41].

### 2.3. Mechanical Measurements with AFM

For assessment of the mechanical properties of the cells, atomic force microscopy (AFM) was used. A Bioscope Resolve AFM (Bruker, Santa Barbara, Goleta, CA, USA) mounted on an Axio Observer inverted fluorescence microscope (Carl Zeiss, Germany) was equipped with a heated stage, and the sample temperature was kept constant at 37 °C. PeakForce QNM-Live Cell probes (PFQNM-LC-A-CAL, Bruker AFM Probes, Camarillo, CA, USA) and short paddle-shaped cantilevers with a pre-calibrated spring constant (average value of 0.1 N/m) were used, and the deflection sensitivity (nm/V) was calibrated from the thermal spectrum using the value of the spring constant [44]. Nanomechanical and topography maps were acquired in fast force–volume (FFV) mode with map sizes from 20 × 20 to 80 × 80 µm and from 32 × 32 to 128 × 128 point-measurements. The force curves (*F-Z* curves) had a vertical ramp distance of 3 μm, a vertical piezo speed of 183 μm/s, and a trigger force of 0.5–1 nN.

Numerical processing of the *F-Z* curves was performed using Python scripts (https://github.com/yu-efremov/ViscoIndent, accessed on 1 October 2023) developed in the previous works [45,46] using Hertz’s and Ting’s models [47] for the elastic and viscoelastic processing, respectively. Young’s modulus (*E_Hertz_*), with the assumptions of Hertz’s theory (“apparent” elastic modulus), was calculated from the approach part of the force curves:

(1) $$F\left(\delta \right)=\frac{4\sqrt{R}}{3(1-\nu^{2})}f_{BEC}\left(\delta \right)E_{Hertz}\delta^{\frac{3}{2}};$$

where *F* is the force acting on the cantilever tip; *δ* is the indentation depth; *ν* is the Poisson’s ratio of the sample (assumed to be time-independent and equal to 0.5); *R* is the radius of the indenter; and *f_BEC_(δ)* is the bottom-effect correction. The latter is a multiplicative analytically derived correction for the indentation contact models that accounts for the finite sample thickness [45,46]. The multiplicative coefficients were taken from the work [48]. The same curves were processed using the viscoelastic model:

(2) $$F\left(t,\;\delta (t)\right)=\frac{4\sqrt{R}}{3\left(1-\nu^{2}\right)}\int_{0}^{t_{1}\left(t\right)}f_{BEC}\left(\delta \right)E(t-\xi )\frac{d\delta^{\frac{3}{2}}}{d\xi }d\xi ;$$

(3) $$t_{1}\left(t\right)=\left\{\begin{array}{c} t_{1}\left(t\right)=t,\;0\leq t\leq t_{m}\;\\ \int_{t_{1}\left(t\right)}^{t}E\left(t-\xi \right)\frac{d\delta }{d\xi }d\xi =0,\;t>t_{m} \end{array}\right.;$$

where *t* is the time initiated at contact; *t_m_* is the duration of the approach phase; *t_1_* is the auxiliary function determined by Equation (3); *ξ* is the dummy time variable required for the integration; and *E(t)* is Young’s relaxation modulus for the selected rheology model. Here, we used the power-law rheology (PLR) model (also known as a springpot in parallel with a dashpot) [49]:

(4) $$E\left(t\right)=E_{0}t^{-\alpha }+\eta \delta_{D}(t)$$

where *E*_0_ is the relaxation modulus at *t* = 1 s (scale factor of the relaxation modulus); *α* is the power-law exponent; *η* is the Newtonian viscous term (with Pa*s units); and *δ_D_* is the Dirac delta function. A larger α value means a larger amount of relaxation; materials exhibit a solid-like behavior at α = 0, and a fluid-like behavior at α = 1. The PLR model described by Equation (3) was successfully used for the description of the cell mechanics in several previous studies [50,51,52,53]. The typical indentation depth was 800–1500 nm. Examples of the force curves together with the model fits are presented in Figure S2.

The topography and cell heights were calculated from the force maps based on the contact point position in individual force curves, after detecting the surface level and performing global tilt correction. The background was then removed by excluding all points with a local height below 200 nm (areas marked with white color on AFM maps). We used the top 50% of each remaining data set over a cell to define the central part, and the lower areas were discarded from the analysis, since the local properties there were highly affected by the high F-actin concentration at the periphery. From the cell datasets, the mean arithmetic values of *E_Hertz_* and viscoelastic parameters were used for further statistical comparison between the samples.

### 2.4. Confocal Microscopy

For the confocal microscopy, cells were cultured in a custom-made 6-well chamber attached to a glass slide. All cell types were seeded in separate wells for 2 days, and then, simultaneously fixed and stained. The samples were fixed in a 4% formaldehyde solution in PBS for 20 min, permeabilized with 0.1% Triton X-100 for 10 min, blocked with 1% bovine serum albumin for 10 min, and stained with Alexa Fluor 488 phalloidin for F-actin and with Hoechst 33342 for the nuclei (Life Technologies, USA). The samples were washed with PBS and mounted with the SlowFade Diamond Antifade Mountant (Invitrogen, Waltham, MA, USA). The fluorescent images (Z-stacks with 0.5 µm step) were acquired using an Olympus IX83-FV3000 (Olympus, Tokyo, Japan) confocal laser scanning microscope with a Plan-Apochromat 100×/1.45 N.A. oil immersion objective. The z-stacks were divided into three sub-stacks (basal, middle, apical), each comprising roughly one third of the stack. Each sub-stack is presented as color-coded z-projections with respect to the scaling shown in the color scale bar. 

### 2.5. ToF-SIMS

For mass spectrometry analysis, the cells were seeded on a 35 mm m-Dish culture dish (Ibidi, Gräfelfing, Germany) with a poly-L-lysine-coated coverslip at the bottom, in an amount of 5 × 10^5^, in complete DMEM (PanEco, Moscow, Russia), and incubated for 24–48 h (37 °C, 5% CO_2_). Then, the coverslips with the cells were washed three times with PBS, and the cells were incubated with 4% paraformaldehyde for 60 min at room temperature for chemical fixation. After that, the cells were washed again three times with PBS and mQ water. Immediately before analysis, the cell samples were pre-dried and dried in a weak current of argon at room temperature. 

A ToF–SIMS 5 mass spectrometer (ION-TOF, Münster, Germany) equipped with a 30 keV Bi_3_^+^ liquid metal ion gun was used for lipid analysis. Mass spectra were recorded both in positive and negative polarities from a randomly selected area of 300 × 300 mm^2^ with a resolution of 64 × 64 pixels. Lipid ion yields were calculated as the intensity of the corresponding peak of interest normalized to the total ion count, and averaged by 64 × 64 pixels. The primary ion dose density did not exceed 5 × 10^11^ ions/cm^2^ for every measurement that was below the static SIMS limit. A low-energy electron flood gun was activated to avoid charging effects. The ion yields from the following key lipid components of the membranes were assessed: phosphatidylcholine (*m*/*z* 224), sphingomyelin (*m*/*z* 264), cholesterol (*m*/*z* 385), and saturated fatty acids, and mono- and polyunsaturated fatty acids.

### 2.6. Statistics

The data are presented as the mean values and the standard deviation (SD). To calculate the statistical significance of the differences in microviscosity and ToF-SIMS data, an ANOVA with a Bonferroni post hoc test was used. *p* ≤ 0.05 was considered statistically significant. For the microviscosity analysis, the number of cells for mean value calculations was 60 in 10 fields of view. For analysis of the AFM data, a Kruskal–Wallis test with Dunn’s multiple comparisons was performed, the data were collected from at least 20 force–volume maps for each cell line. Statistical analysis of the ToF-SIMS data was performed using SurfaceLab 7 software (ION-TOF, Münster, Germany). Asterisks on the graphs indicate significance in the analysis: * *p* ≤ 0.05, ** *p* ≤ 0.01, *** *p* ≤ 0.001, **** *p* < 0.0001.

## 3. Results

### 3.1. Measurement of Membrane Microviscosity of Colorectal Cancer Cell Lines via FLIM

**Figure 1:**
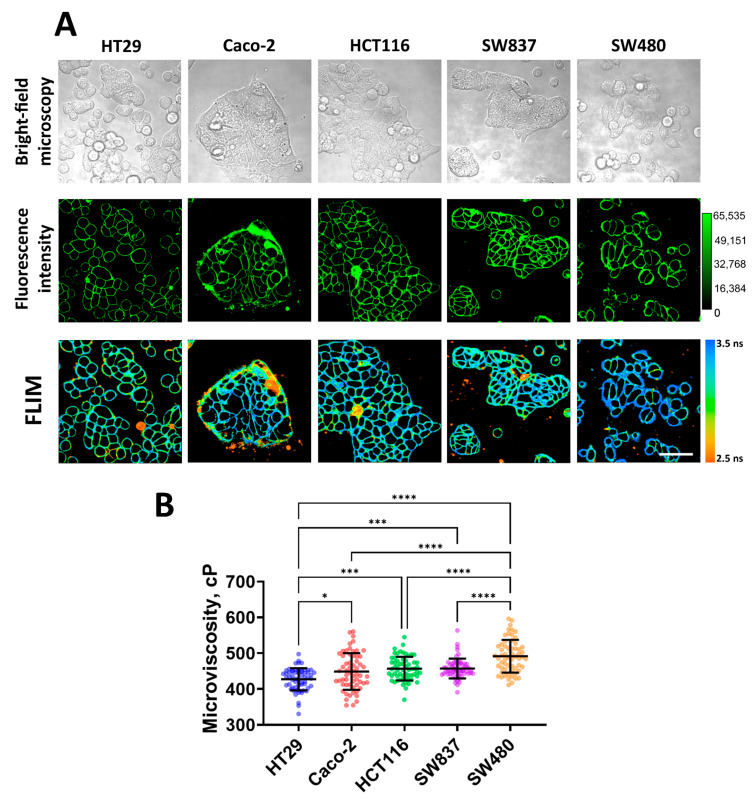
Imaging of membrane microviscosity in colorectal cancer cell lines. (**A**) Representative bright-field, fluorescence intensity, and FLIM images of different cell lines. Scale bar, 50 μm. BODIPY2 (4.5 μM) was used as a viscosity-sensitive probe. Ex. 850 nm, reg. 500–550 nm. (**B**) Quantification of microviscosity of plasma membranes in cancer cells. Mean ± SD, *n* = 60 cells. Scatter dot plot displaying the measurements for individual cell membrane (dots) and the means (horizontal lines) of microviscosity of different cell lines. Asterisks on the graphs indicate significance in the analysis: * *p* ≤ 0.05, *** *p* ≤ 0.001, **** *p* < 0.0001.

Using FLIM with the molecular rotor BODIPY 2, plasma membrane microviscosity was measured for five cell lines of human colorectal cancer (HT29, Caco-2, HCT116, SW 837, and SW 480, in order of increasing mesenchymal phenotype and migratory activity). We observed efficient fluorescent staining of the plasma membranes in all cell lines used (Figure 1A). As previously established, BODIPY dyes stain the lipid tail region of lipid bilayers [40], thus providing a true representation of comparative viscosity between various lipid bilayers [54].

Quantitative analysis showed differences in the values of membrane microviscosity among cells of different lines (Figure 1B). It was found that HT29 cells have the most fluid membranes. The fluorescence lifetime of the rotor in their membranes was 2.95 ± 0.10 ns, which corresponded with a microviscosity value of 427 ± 30 cP. Caco-2, HCT116, and SW837 cells have slightly more viscous membranes compared to HT29, and their values of microviscosity were 448 ± 51 cP (3.03 ± 0.17 ns), 456 ± 33 cP (3.06 ± 0.11 ns), and 457 ± 27 cP (3.06 ± 0.09 ns), respectively. The highest microviscosity was detected for the membranes of SW480 cells—the fluorescence lifetime of the rotor in the membrane was 3.17 ± 0.14 ns, which corresponded with a microviscosity 491 ± 45 cP. The obtained microviscosity values are similar to those obtained in previous studies conducted on cancer cells using the FLIM technique [38,39,42].

It should be noted that selected cell lines have different migratory capacities due to different epithelial–mesenchymal phenotypes [55]. Of them, HT29 cells are characterized as having an epithelial morphology (spherical or polygonal shape, small size, tight junctions between cells) and a low level of migration activity, and SW480 cells have a mesenchymal-like phenotype (elongated spindle-like or more rounded shape, fine protrusions) and the highest migratory capacity. Other cell lines have intermediate epithelial/mesenchymal statuses. Therefore, low microviscosity of cancer cell membranes correlated with low intrinsic motility, and vice versa, a high microviscosity was observed for more motile cells.

**Figure 2:**
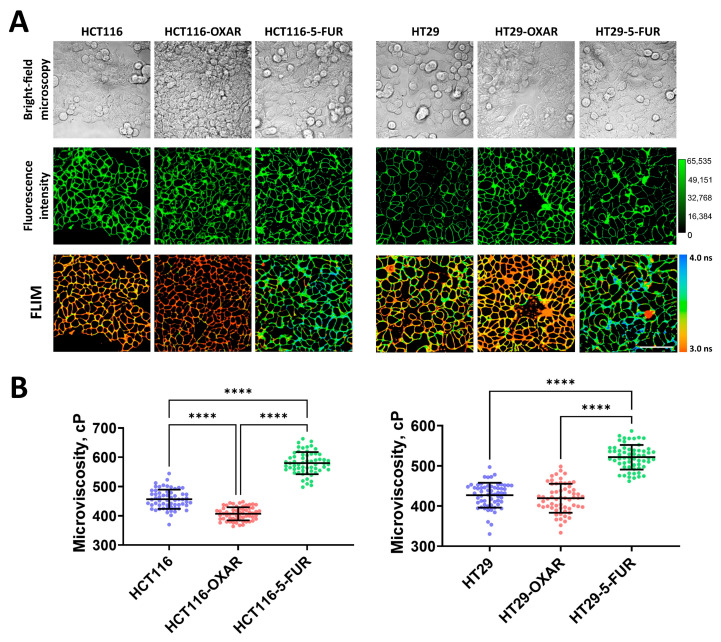
Membrane microviscosity in colorectal cancer cells resistant to oxaliplatin and 5-fluorouracil. (**A**) Representative bright-field microscopic, fluorescent, and FLIM images of colorectal cell sublines. Scale bar, 50 μm. BODIPY 2 (4.5 μM) was used as a viscosity-sensitive probe. Ex. 850 nm, reg. 500–550 nm. (**B**) Quantification of microviscosity of plasma membranes in resistant cancer cells. Mean ± SD, *n* = 60 cells. Scatter dot plot displaying the measurements for individual cell membranes (dots) and the means (horizontal lines) of microviscosity of different cell lines. Each dot is the mean value for 60 cells in 10 fields of view. Asterisks on the graphs indicate significance in the analysis: **** *p* < 0.0001.

To further determine whether the drug resistance of cancer cells changes the fluid properties of their membranes, we measured microviscosity in oxaliplatin- and 5-FU-resistant cells (Figure 2). It was found that in most resistant cell sublines, the microviscosity of the plasma membranes was different from that of the parental line. Both 5-FU-resistant sublines had significantly more viscous membranes than their sensitive controls: in HCT116-5-FUR cells, the rotor fluorescence lifetime was 3.45 ± 0.11 ns, which corresponded with 579 ± 37 cP, and in HT29-5-FUR cells, the lifetime was 3.27 ± 0.09 ns and microviscosity was 521 ± 30 cP. The cells that were resistant to oxaliplatin showed a tendency toward the fluidization of plasma membranes. In HCT116-OXAR cells, microviscosity was statistically lower than in the control (407 ± 22 cP), while in HT29-OXAR, only slight fluidization was observed (419 ± 36 cP), but these differences were statistically insignificant.

Therefore, the acquisition of drug resistance by colorectal cancer cells can result in alterations in their membranes’ viscous properties; however, this depends on the drug to which the resistance was acquired.

### 3.2. Assessment of Mechanical Properties of Colorectal Cancer Cell Lines via AFM

**Figure 3:**
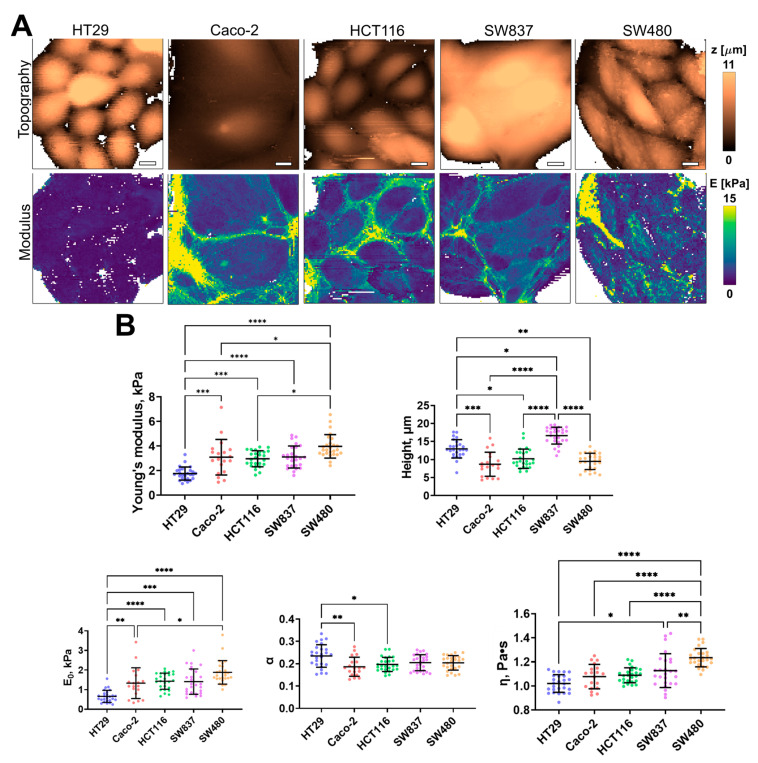
Nanomechanical analysis of colorectal cancer cells via AFM. (**A**) Examples of AFM fast force–volume maps, topographies, and the corresponding apparent Young’s modulus maps. Scale bar, 10 μm. (**B**) Quantification of the apparent Young’s modulus and viscoelastic parameters of colorectal cancer cells. Scatter dot plot displaying the measurements for individual force maps (dots represent the mean value taken from the pixels above 50% of the maximum height, as defined by the corresponding topography images) and the mean ± SD (horizontal lines) of microviscosity of different cell lines. Asterisks on the graphs indicate significance in the analysis: * *p* ≤ 0.05, ** *p* ≤ 0.01, *** *p* ≤ 0.001, **** *p* < 0.0001.

Using AFM, the cell height, apparent Young’s modulus (stiffness), and several viscoelastic parameters (E_0_, η, and α) were assessed for the same five cell lines of human colorectal cancer. The distribution of these parameters, taken from fast force–volume maps from different cell lines, is presented in Figure 3. The HT29 and SW837 cells were the tallest, while SW480 and CaCo-2 cells were more spread out. SW480 and SW837 demonstrated the highest mean stiffness, while HT29 cells demonstrated the lowest stiffness. A similar trend was observed in two viscoelastic parameters, E_0_ and η, while the power-law exponent α demonstrated close to the opposite behavior, with HT29 cells having the largest value. As shown in previous studies, changes in the viscoelastic parameters are often related, and stiffer cells have higher values of E_0_ and lower values of α [46]. Overall, the values of the apparent Young’s modulus (1–4 kPa) and of the viscoelastic parameters (E_0_ ≈ 0.5–2 kPa; α ≈ 0.15–0.25; η ≈ 0.9–1.4 Pa∙s) are close to those obtained in previous studies on cancer and epithelial cell lines [46,56,57].

**Figure 4:**
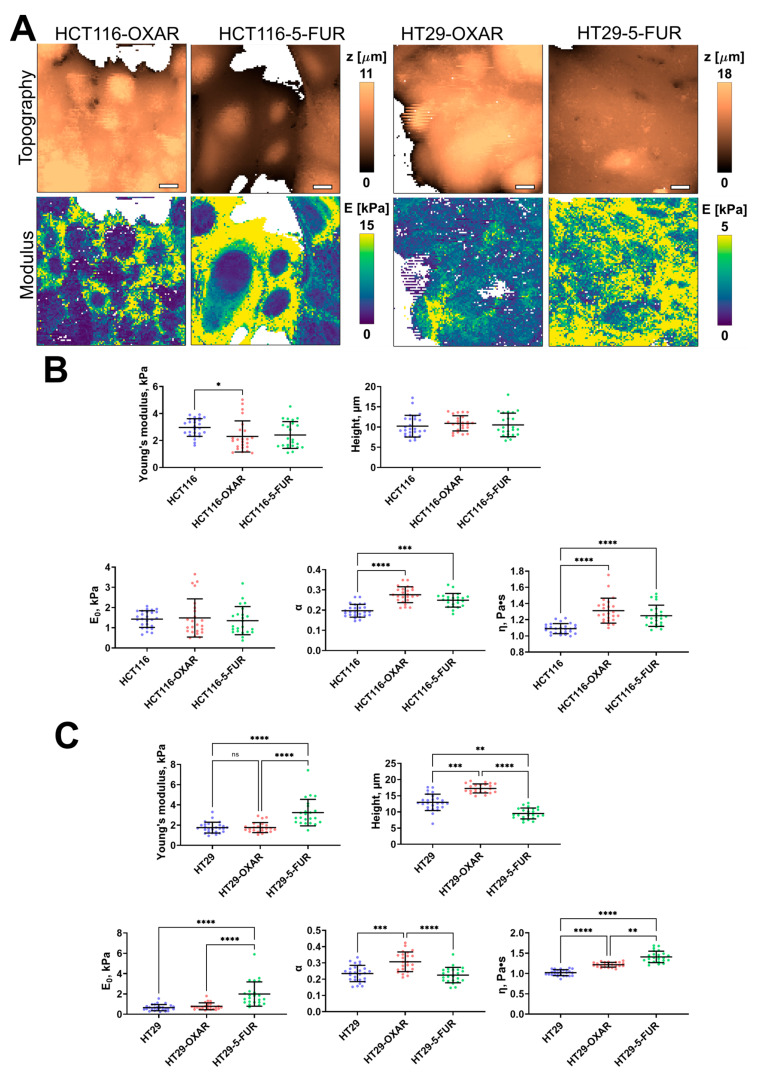
Nanomechanical analysis of colorectal cancer cells resistant to oxaliplatin and 5-fluorouracil. (**A**) Examples of AFM fast force–volume maps, topographies, and the corresponding apparent Young’s modulus maps. Scale bar, 10 μm. (**B**) Quantification of the apparent Young’s modulus and viscoelastic parameters of resistant cancer cells. (**C**) Quantification of the apparent Young’s modulus and viscoelastic parameters of HT29-OXAR and HT29-5-FUR cells. Scatter dot plot displaying the measurements for individual force maps (dots represent the mean value taken from the pixels above 50% of the maximum height, as defined by the corresponding topography images) and the mean ± SD (horizontal lines) of different cell lines. Asterisks on the graphs indicate significance in the analysis: * *p* ≤ 0.05, ** *p* ≤ 0.01, *** *p* ≤ 0.001, **** *p* < 0.0001.

The 5-FU- and oxaliplatin-resistant cell lines demonstrated significant changes only in some of the viscoelastic parameters (Figure 4). The apparent Young’s modulus of HCT116-OXAR was significantly lower in comparison with the control cells, while the power-law exponent and viscosity of both HCT116-OXAR and HCT116-5-FUR were significantly higher. The apparent Young’s modulus, E_0_, and viscosity of HT29-5-FUR and HT29-OXAR cells were significantly higher than the corresponding parameters of the control HT29 cells. The height and the power-law exponent of HT29-OXAR cells were significantly higher, while the height of HT29-5-FUR cells was significantly lower.

### 3.3. Actin Cytoskeleton of Colorectal Cancer Cell Lines Visualized via Confocal Microscopy

**Figure 5:**
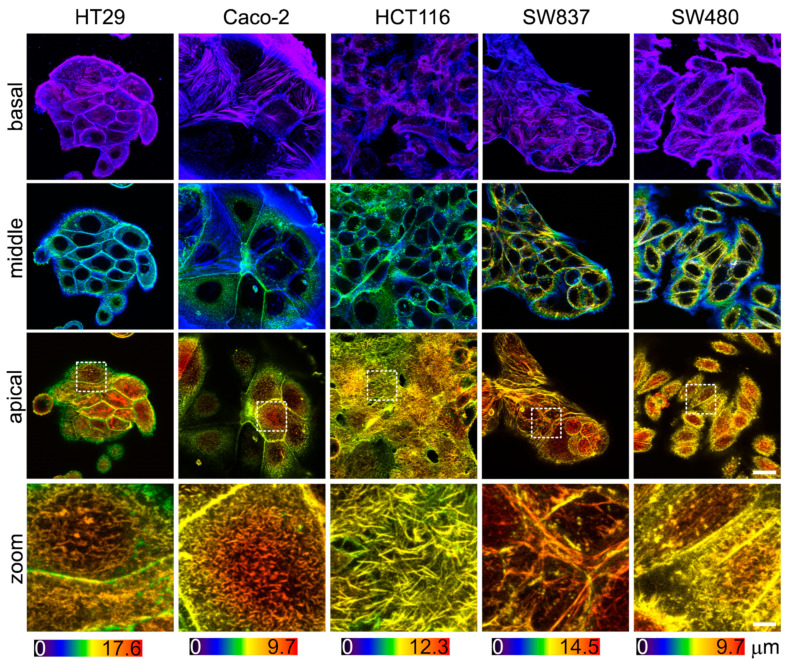
The actin cytoskeleton of colorectal cancer cells visualized via confocal microscopy. Color-coded z-projections of the F-actin staining divided into basal, mid-height, and apical regions. Bottom row: zoomed-in areas of the white square of the apical region, demonstrating large number of microvilli in HCT116 cells. Scale bar, 20 μm (4 μm for zoomed-in areas).

Since the viscoelastic properties of cells are largely determined by the sub-membrane actin cytoskeleton, an assessment of its morphology was performed. The actin cytoskeleton was analyzed via confocal microscopy in fixed and stained samples of cells (Figure 5). In agreement with the AFM data, HT29 and SW837 cells were the tallest, and Caco-2 and SW480 cells were the most spread out. Stress fibers were barely observed in the basal plane of all cell types and were lacking in the apical region, which is in agreement with previous studies of cancer cell cytoskeletons [20,58]. HCT116 cells were densely covered in microvilli, while other cells had fewer microvilli (Figure 5, zoomed-in areas of apical regions). Due to the presence of actin-containing microvilli, it was impossible to assess the actin density in the cell cortex. Overall, the intensity of the stained actin in the apical region was similar for all cell types.

### 3.4. Assessment of Lipid Profile of Colorectal Cancer Cell Lines via ToF-SIMS

**Figure 6:**
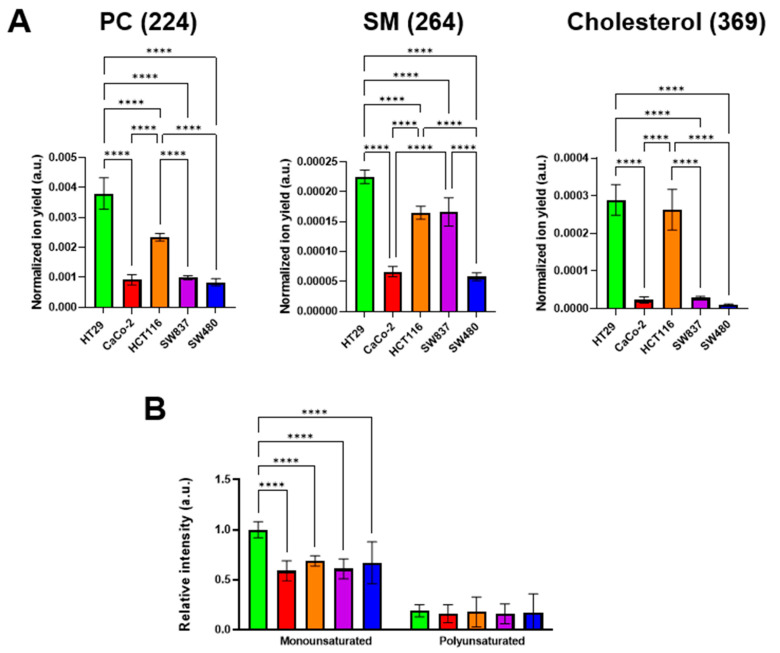
ToF-SIMS analysis of membrane lipid composition in colorectal cancer cell lines. (**A**) Phosphatidylcholine (PC) (*m*/*z* 224), sphingomyelin (SM) (*m*/*z* 264), and cholesterol (*m*/*z* 385) ion yields were obtained in positive ions. (**B**) Mono- and polyunsaturated fatty acids were obtained in negative ions. Data on fatty acids were normalized to the saturated fatty acids signal of the corresponding sample. Mean ± SD. Asterisks on the graphs indicate significance in the analysis: **** *p* < 0.0001.

It is known that the viscous properties of cell membranes are largely determined by the qualitative and quantitative lipid composition. Among the lipid components, cholesterol and sphingomyelin (SM) make the membrane more viscous, whereas phosphatidylcholine (PC) and mono- and polyunsaturated fatty acids fluidify it [34,59]. It was found that different colorectal cancer cell lines have different membrane lipid profiles. The highest signals of cholesterol, SM, and monounsaturated fatty acids were recorded in HT29 cells. At the same time, PC ion yield was also the highest in this cell line (Figure 6). The lowest cholesterol, SM, and PC signals were detected in SW480 cells. Relatively low signals of these components were recorded in CaCo-2 cells. The signals of polyunsaturated fatty acids showed no differences between the cell lines used.

We assessed the relationships between the signals of specific membrane components and microviscosity values (Figure S3). A moderate negative correlation with membrane microviscosity was obtained for PC (Pearson *r* = −0.72), SM (*r* = −0.72), cholesterol (*r* = −0.62), and monounsaturated FAs (*r* = −0.59). The correlation of polyunsaturated FAs and microviscosity was weak (*r* = −0.42). While negative correlations of PC and unsaturated FAs with membrane viscosity was an expected result, the observation of such a correlation for SM and cholesterol is rather unusual. The reason for these discrepancies could be the fact that PC and phospholipids with unsaturated FAs in their tails constitute mainly the bulk part of the membrane bilayer, whereas SM and cholesterol are also included in the membrane rafts—the highly functional, dynamic, relatively ordered domains. Using molecular rotor BODIPY 2, the microviscosity of only the bulk part of membranes was measured, but ToF-SIMS provided the integral data across the whole membrane. 

The obtained data on the membrane lipid composition of colorectal cancer cell lines suggest that the microviscosity of the lipid bilayer is most likely determined not by a single constituent, but by a complex network of different ones.

### 3.5. Comparison of Membrane Microviscosity and Cell Mechanical Properties of Colorectal Cancer Cell Lines

**Figure 7:**
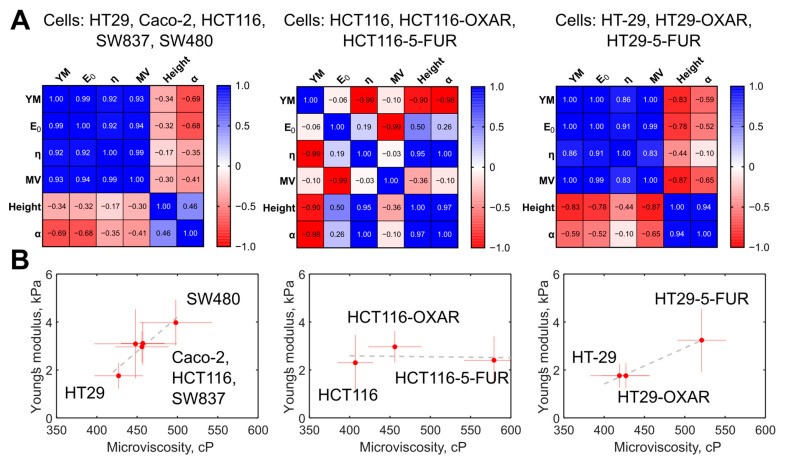
The correlation between the measured mechanical parameters of the colorectal cancer cells. (**A**) Heatmaps of the correlation matrix. Each variable is represented by a row and a column, and the cells show the correlation between them. The values of Pearson’s correlation coefficient (r) are presented in the cells and color coded according to the color scale bar. The analysis was performed for the groups of the five studied colorectal cancer cells, for the group of HT29, HT29-OXAR, and HT29-5-FUR cells, and for the group of HCT116, HCT116-OXAR, and HCT-116-5-FUR cells. YM = Young’s modulus; MV = microviscosity. (**B**) Graphs of Young’s modulus versus membrane microviscosity demonstrating the presence of correlations for the five studied colorectal cancer cells, for the group of HT29, HT29-OXAR, and HT29-5-FUR cells, but not for the group of HCT116, HCT116-OXAR, and HCT-116-5-FUR cells.

The membrane microviscosity, assessed via FLIM, and cell mechanical properties, assessed via AFM, were compared (Figure 7A). First, we observed a strong positive correlation between a group of the parameters measured via AFM, namely, Young’s modulus, E_0_, and η, with Pearson *r* > 0.92 (*p* < 0.05). For all the parameters from that group, a strong positive correlation with membrane microviscosity was found (*r* > 0.93, *p* < 0.05). A weak negative correlation was observed between the named mechanical parameters and the cell height and the power-law exponent α (*r* = −0.35… −0.69, *p* > 0.05). On the plot of the mean values of Young’s modulus versus the mean values of microviscosity (Figure 7B), a cluster of cells with similar parameters can be distinguished (Caco-2, HCT116, SW837), while HT29 cells had substantially lower values and SW480 had substantially higher values of these parameters.

For the chemoresistant cells, we observed a similar positive correlation between Young’s modulus and membrane microviscosity (*r* > 0.83, *p* < 0.05) in the case of HT29, HT29-OXAR, and HT29-5-FUR cells, but not HCT116, HCT116-OXAR, and HCT-116-5-FUR cells.

Correlations between cell mechanical parameters (cell height, stiffness, and power-law exponent) were found in previous AFM studies on different cell types, as well [60,61,62], but membrane microviscosity was directly compared with cortex mechanics quantitatively on a range of cell lines for the first time in the present study.

## 4. Discussion

The mechanical characteristics of both the cell plasma membrane and the sub-membrane cortex play an important role in cell functioning, and their changes are often associated with various pathologies, including carcinogenesis. Studies of cellular biomechanics are especially relevant in the context of the migration and invasion of cancer cells, as these abilities underlie tumor progression. Here, by performing a comprehensive analysis of the viscoelastic properties of human colorectal cancer cells at the cellular and subcellular scales, we found a strong correlation between cell stiffness, assessed via AFM, and the microviscosity of the plasma membrane, assessed via FLIM. Of five cell lines, the more motile cells of the mesenchymal phenotype had a higher stiffness and a more viscous membrane.

Previously, changes in the biophysical properties of cancer cells associated with their migratory capacity were observed in various studies. Most studies conducted to date have found that individual cancer cells are softer than healthy cells, as reviewed in [29,63,64]. However, there is less consensus about differences in the stiffness of cancer cells with different invasive capacities. It was shown that more invasive cancer cells either soften [65,66,67] or stiffen [68,69,70,71] during tumor progression. In part, the observed discrepancies can be caused by a huge variability of cancer cells and signaling pathways involved in the invasion process [72]. Here, we observed higher stiffness (apparent Young’s modulus and E_0_) of the cells with a mesenchymal-like phenotype and the highest migratory capacity (SW480) and lower stiffness in the cells with epithelial morphology and a low level of migration activity (HT29). The cell height and the power-law exponent were larger in the softer HT29 cells, which is in agreement with the previous studies [60,62]. The Newtonian viscosity parameter η of the applied viscoelastic model demonstrated a positive correlation with stiffness, which could be related to the viscosity of the cytoplasm near the cortex, which is also probed at the indentation depths applied here (800–1500 nm, typically). But currently, there are no other data to support this conclusion.

The invasive potential of cancer cells strongly depends on the rigidity of the cells as a whole. This parameter is regulated not only by the mechanical properties of the cytoskeleton, but also by the physical properties of the membrane, in particular, its viscosity. The data on the membrane viscosity characteristics of cells with different levels of migration activity are poor. For example, Kaur et al. examined the role of membrane fluidity in the carcinogenic transformation of colonic epithelial cells. Using the fluorescence polarization method with a 1,6-diphenylhexatriene (DPH) probe, it was shown that membrane fluidity increases early upon the chemical induction of carcinogenesis [73]. In the works by Angelucci et al. on a panel of human breast cancer cell lines with different migratory activity, using the fluorescent dye Laurdan, it was found that minimally invasive MCF-7 cells with a high expression of E-cadherin had more fluid membranes than highly invasive MDA-MB-231 cells [74,75], which is consistent with our results. In a panel of cell lines in our study, HT29 cells had the highest expression of E-cadherin and the most fluid membranes, while vimentin and integrin αV were highly expressed in SW480 cells that had the most viscous membranes [55]. The fluidity of cell membranes allows for the lateral mobility of proteins, including E-cadherin, along the cell surface [76]. E-cadherin is a transmembrane glycoprotein involved in Ca^2+^-dependent cell–cell adhesion [77]. It was suggested that membrane fluidity mediates lateral interaction between cadherin bonds—high fluidity facilitates the movement of E-cadherin within the plasma membrane and the formation of cell–cell junctions. M. Ozawa and R. Kemler showed that an increase in membrane viscosity can prevent E-cadherin clustering [78]. The work by Tsai and Kam showed that lateral mobility of E-cadherin enhances recognition, allowing for the functional activation of downstream signaling processes at lower surface concentrations than their immobile counterparts [76]. Therefore, the differences in membrane viscosity between the different cell lines used in our study can be associated with alterations in cell–cell adhesion.

Membrane fluidity regulates not only E-cadherin-, but also E-selectin-mediated adhesion. E-selectin is a vascular endothelial molecule that initiates circulating tumor cell recruitment at metastatic sites. By modulating cholesterol levels in cell membranes, Mohammadalipour et al. found that lowering cholesterol levels, which increased membrane fluidity, reduced the adhesion of non-small-cell lung cancer cells. At the same time, cholesterol depletion did not significantly affect the membrane viscosity and adhesion of small-cell lung cancer cells [79]. 

Cancer cell invasion typically requires cell adhesion to the extracellular matrix via integrins—the cell–matrix adhesion receptors. The relationship between integrin expression and tumor cell membrane viscosity has been poorly explored. For example, it was shown that the expression of integrin αvβ3 increases the invasiveness of MDA-MB-231 cancer cells by increasing cellular rigidity and enhancing the dynamics of cytoskeletal remodeling (cellular fluidity) [80]. Gopalakrishna et al. discussed that an increase in cholesterol content in plasma membranes and, consequently, a decrease in its fluidity in rat fibroblasts caused a significant increase in the clustering of α5β1 integrin molecules in focal adhesions, their adhesion to the cell-binding domain of fibronectin, and their association with the cytoskeletal protein talin [81].

Interestingly, different cell lines of the same histogenesis were characterized by dramatically different membrane lipid profiles according to our ToF-SIMS data. Increased signal intensity of phospholipids was recorded in the HT29, HCT116, and SW837 cell lines, and the highest cholesterol level was detected in HT29 and HCT116 cells, while the level of polyunsaturated fatty acids was the same in all lines. A significantly higher level of monounsaturated fatty acids was found in HT29 cells. The study by Ohmori et al. demonstrated a relationship between the tumorigenic properties of HT29 cells and the amount of monounsaturated fatty acids. It has been shown that supplementation with elaidic acid, the trans form of oleic acid, significantly increased the survival of CT26 and HT29 colorectal cancer cells [82]. The greater metastasis potential caused by elaidic acid was explained by the enhancement of growth and stemness of HT29 cells through the activation of the epithelial growth factor receptor pathway in lipid rafts [83]. In addition, elaidic acid enhanced CT26 and HT29 cell proliferation and conferred resistance to 5-fluorouracil [82]. Previously, increased levels of phospholipids, sphingomyelin, and cholesterol were observed in colon cancer tissue compared with normal colon mucosa [84]. In addition, monounsaturated oleic acid content was decreased in cancer tissues, while the levels of saturated fatty acids and polyunsaturated fatty acids, which are the major components of polar lipids, were increased [85]. Correlative analysis of membrane lipid composition and microviscosity showed that the specific components correlate with the fluid properties of the bulk membrane bilayer (a decrease in the content of mono- and polyunsaturated fatty acids correlates with an increase in microviscosity). Meanwhile, microviscosity values lay in a narrow range (420–490 cP), although the differences between different cell lines were statistically significant. Lipidome flexibility of biological membranes in the context of their physical homeostasis was reported in several studies (reviewed in [86]). 

The interplay between the mechanical properties of the plasma membrane and the cytoskeleton is a long-standing question in cell biomechanics [1,2,87]. In particular, tether pulling experiments were established as an effective tool to study this interplay [10,11,12,13]. This method consists in extracting tethers (lipid membrane tubes) by applying a force on the membrane via a small probe (e.g., an AFM probe, or a microsphere controlled by optical tweezers). This method allowed for the evaluation of effective membrane tension, which was shown to be affected strongly by membrane–cortex interactions [10,11,88,89,90,91], and effective membrane viscosity, supposedly originating from viscous friction between the cytoskeleton and membrane and from the flow of the membrane around the transmembrane proteins [92,93,94]. Higher viscosity was measured upon cholesterol enrichment, and lower viscosity was measured upon depletion, but cholesterol depletion strengthened plasma membrane–cytoskeleton adhesion [94]. By pulling two tethers simultaneously at different positions on the same cell, it was revealed that that propagation of membrane tension is largely suppressed in intact cells due to the flow resistance from cytoskeleton-bound transmembrane proteins [15,16]. Another work showed a positive correlation between membrane-proximal actin density and membrane tension [95]. It should be noted that the viscosity estimated by the molecular rotors is mainly related to the viscosity of the hydrophobic part of the lipid bilayer, and at the same time, is very sensitive even to small changes in bilayer properties [40,96]. 

In our work, to the best of our knowledge, the fluidity (microviscosity) of the membrane was directly compared with cell deformability (AFM) in the same several cell lines for the first time. A strong positive correlation was found between cell stiffness (apparent Young’s modulus, viscoelastic modulus E_0_)—which, due to the used AFM technique, is mostly related to the actomyosin cortex—and membrane microviscosity. Previously, in the work by Silva et al., both membrane fluidity (qualitatively, via generalized polarization of the environment-sensitive fluorescent probe Laurdan), and cell elasticity (via AFM) were assessed for the same pancreatic cancer cells before and after the silencing of Aquaporin-3 (AQP3) and Aquaporin-5 (AQP5) [97]. Silenced AQP5 and AQP3/5 cells showed both lower membrane viscosity and a lower stiffness than their control, which agrees with the current study, hypothetically due to alterations in the cytoskeleton after silencing. In line with the observed trend, previous data indicate that the mesenchymal stromal cells, which are significantly stiffer than the cancer cells used here (5–10 kPa, measured via AFM [98]) also have higher membrane microviscosity (≈1400 cP at 21 days of cultivation, measured via FLIM [99]).

**Figure 8:**
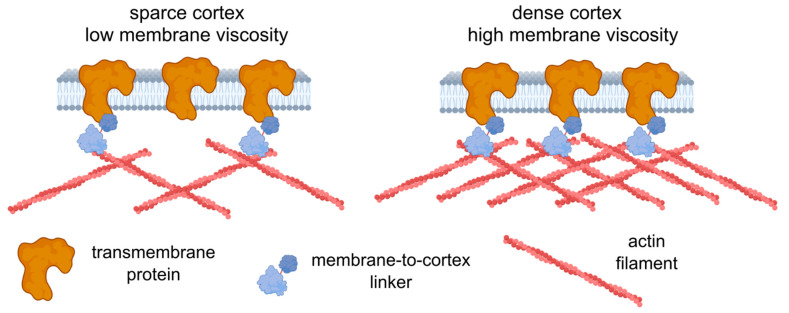
A possible model relating cell deformability and plasma membrane fluidity. An increase in cortex density potentially increases both cell stiffness and membrane microviscosity (decreases fluidity) via membrane-to-cortex linkers and the immobilization of transmembrane proteins. The figure was created using BioRender.com.

Further experiments are required to establish the origin of the observed correlation, yet we can speculate on the possible reasons. First, the increase in the concentration of transmembrane proteins should lead to an increase in the effective membrane microviscosity, since such proteins can be considered obstacles to lipid flow. There are theories describing such dependencies [1,100], but the quantitative description is beyond the scope of the present work. The immobilized transmembrane proteins can affect viscosity even more substantially, since they produce stable obstacles. Immobilization can be achieved via dynamic direct or indirect (e.g., via membrane-to-cortex linkers) linkage with the underlying actin cortex [27,88]. In turn, higher cortical stiffness can be related to the higher density of the actin cortex [101], which consequently allows for more attachment points between the transmembrane proteins and the cortex. Therefore, an increase in the actin density can affect both cell deformability (directly) and membrane viscosity (indirectly via the increasing fraction of the attached proteins) (Figure 8). Alternatively, an increase in the expression of the membrane–cortex linker proteins (ezrin, radixin, moesin (ERM)) may cause a decrease in membrane fluidity [102]. High levels of Ezrin were found to be associated with metastatic behavior in various types of cancer [103]. However, the observed correlation might not be absolute, as was shown here in chemoresistant HCT116 cells, and other factors (including lipid composition) can play a significant role in the maintenance of membrane fluidity. 

A limitation of our study is that the measurements of mechanical properties was only carried out for cancer cells, without comparisons with the appropriate normal cells with mesenchymal and epithelial phenotypes (e.g., normal colon fibroblasts and normal colon epithelium). The study of fluidity and elasticity of normal cells and comparisons with cancer will be the subject of our future research. The other directions that require attention are an analysis of transmembrane and membrane-to-cortex linker proteins, as well as of the local actin cortex density in the same cell types.

## 5. Conclusions

Most studies in the field of cellular mechanics are focused on cytoskeleton and membrane proteins, primarily adhesion proteins and their associated signaling pathways, while the roles of the biophysical and chemical parameters of the cell membrane have been studied relatively poorly. Here, using a combination of several techniques—AFM, FLIM microscopy with fluorescent molecular rotor BODIPY 2, confocal microscopy, and ToF-SIMS—we made an attempt to find any correlations between the microviscosity of the plasma membrane, its lipid composition, and the mechanical properties of cells in a panel of colorectal cancer cell lines. Our results suggest that the mechanical parameters of cells, determined mainly by assessing the sub-membrane cytoskeleton, and viscous properties of the membrane bilayer are linked to each other. The mechanisms of this linkage have yet to be established, but it is clear that they are crucial for cancer cell motility and adhesion. Since the mechanical activity of cells is accompanied by changes in their viscoelastic properties, their cooperative measurements can help to better understand the mechanisms of cancer progression and find ways to prevent cancer cell dissemination. Our future work will continue to explore the interconnections between the physical properties of membranes and biomechanics of whole cells in cancer, with more precise attention paid to EMT, cell migration, and modifications in more complex multicellular systems.

## Data Availability

The data presented in this study are available on request from the corresponding authors.

## Supplementary Materials

The following supporting information can be downloaded at: https://www.mdpi.com/article/10.3390/cells12212583/s1, Figure S1: Representative decay curves of BODIPY 2 in plasma membranes of panel colorectal cancer cells. The fit with a monoexponential decay model is shown with black curves; Figure S2: Representative examples of the force curves together with the model fits for all cell lines studied. Black curve—experimental data; red curve—Hertz’s model fit; blue dashed curve—Ting’s/power-law rheology model fit; Figure S3: The correlation between the measured microviscosity and lipid components of colorectal cancer cells. Graphs of membrane microviscosity versus different lipid components demonstrating the presence of a correlation for the five studied colorectal cancer cells.
