# Supplementary material for: Correlation of Plasma Membrane Microviscosity and Cell Stiffness Revealed via Fluorescence-Lifetime Imaging and Atomic Force Microscopy
Source: Cells. 2023 Nov 6;12(21):2583. doi: 10.3390/cells12212583 (PMC10650173; doi:10.3390/cells12212583)
Supplement: Supplementary file 1 [file cells-12-02583-s001.zip › cells-2643772-supplementary.pdf]

Supplementary Information for

Correlation of the Plasma Membrane Microviscosity and Cell Stiffness Revealed by Fluorescence Lifetime Imaging and Atomic Force Microscopies

Yuri M. Efremov, Liubov Shimolina, Alexander Gulin, Nadezhda Ignatova, Margarita Gubina, Marina K. Kuimova, Peter S. Timashev, Marina Shirmanova

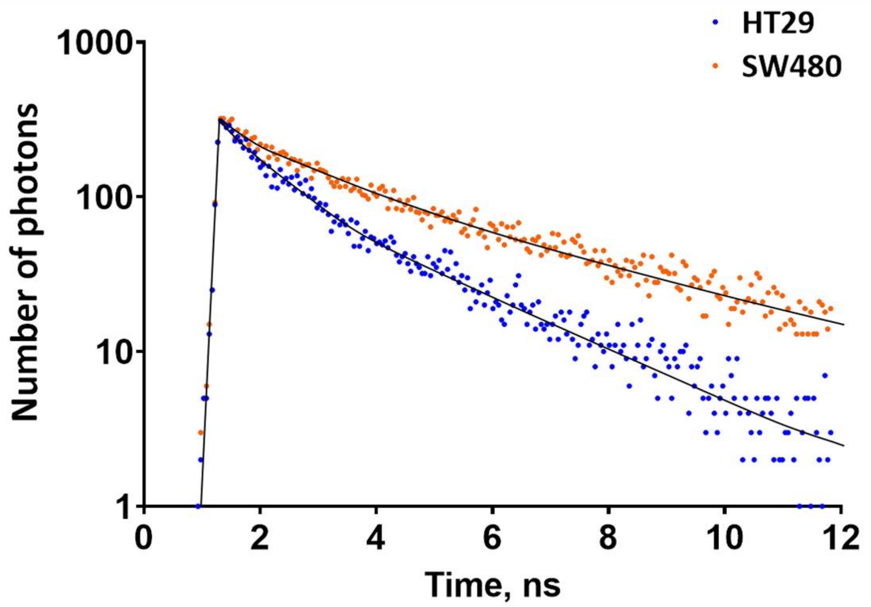

Figure S1. Representative decay curves of BODIPY 2 in plasma membranes of panel colorectal cancer cells. The fit with a monoexponential decay model is shown with black curves.

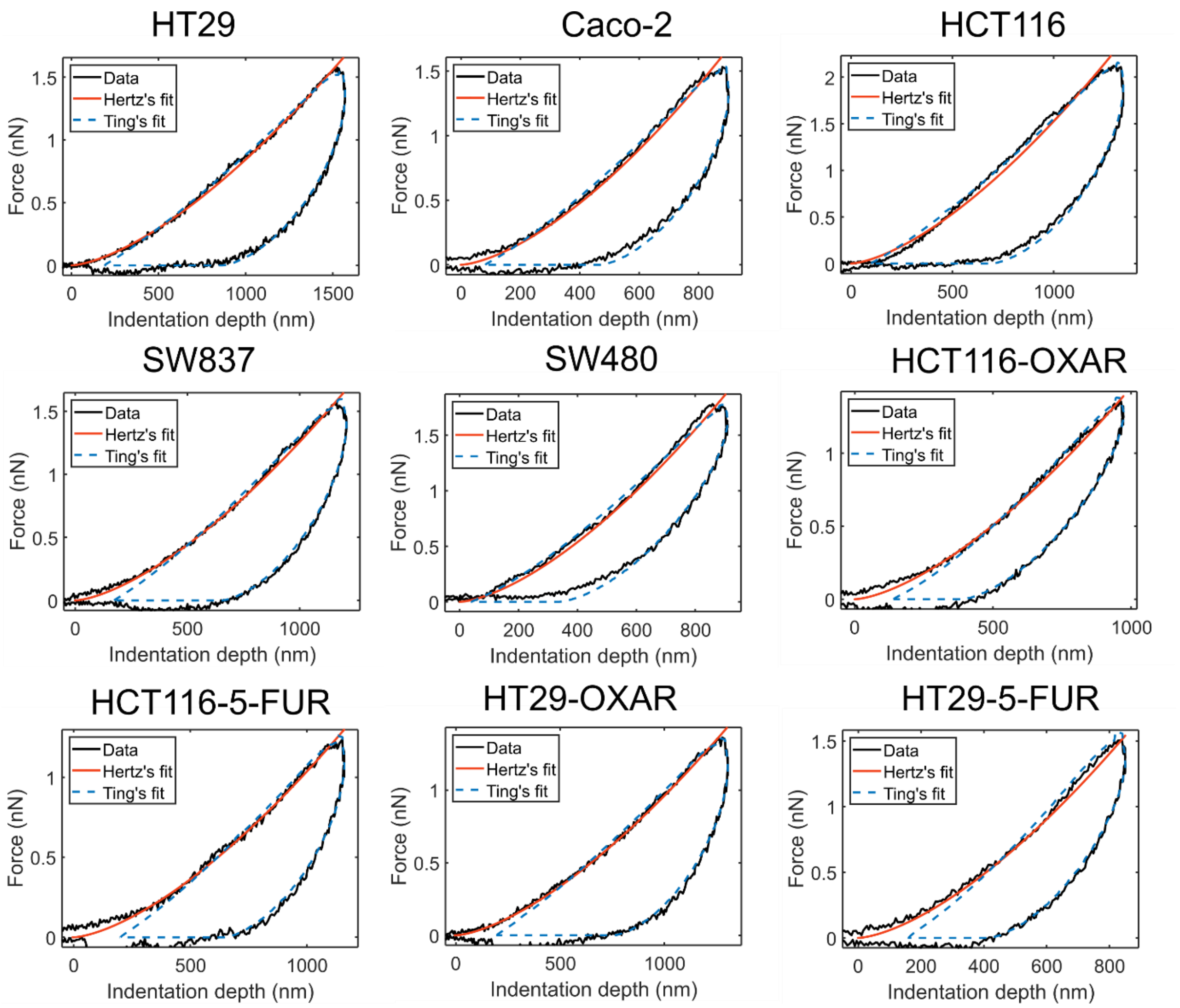

Figure S2. Representative examples of the force curves together with the model fits for all cell lines studied. Black curve – experimental data; red curve – Hertz's model fit; blue dashed curve – Ting's/power-law rheology model fit.

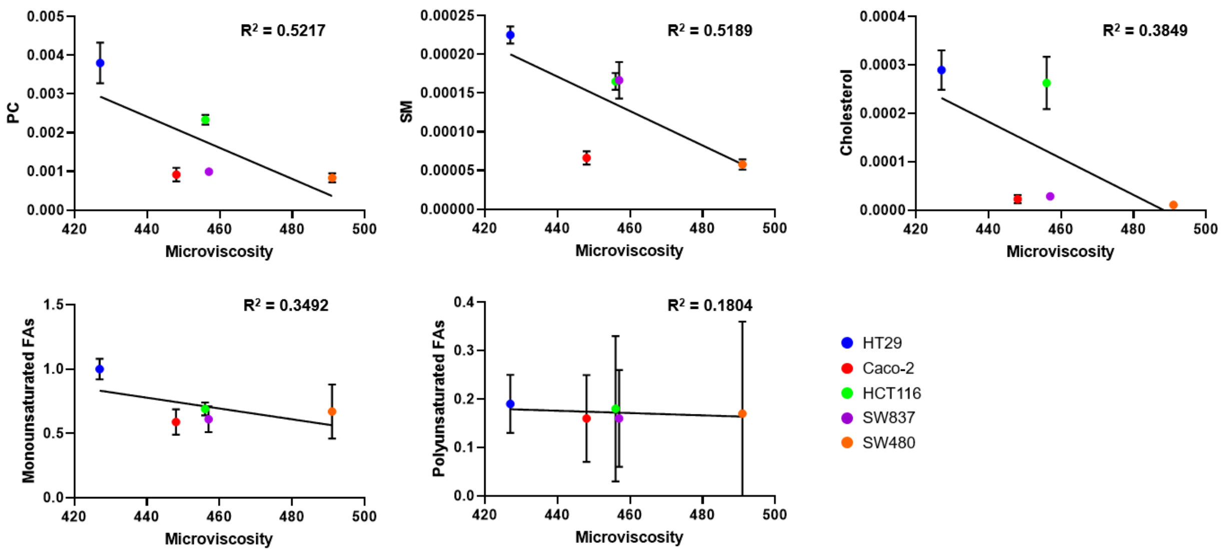

Figure S3. The correlation between the measured microviscosity and lipid components of the colorectal cancer cells. Graphs of the membrane microviscosity versus different lipid components demonstrating presence of the correlation for the five studied colorectal cancer cells.
